# Identification of a novel SPTB gene splicing mutation in hereditary spherocytosis: a case report and diagnostic insights

**DOI:** 10.3389/fgene.2024.1522204

**Published:** 2025-01-30

**Authors:** Xiaobing Li, Tingqiang Zhang, Xuemei Li, Li Wang, Qian Li, Qianqian Liu, Chengyin He, Li Zhang, Yongsheng Liu, Junling Tang

**Affiliations:** ^1^ Science and Technology Industry Development Center, Chongqing Medical and Pharmaceutical College, Chongqing, China; ^2^ Department of Occupational Disease and Poisoning Medicine, The First Affiliated Hospital of Chongqing Medical and Pharmaceutical College, Chongqing, China; ^3^ Laboratory of Toxicology, The First Affiliated Hospital of Chongqing Medical and Pharmaceutical College, Chongqing, China; ^4^ Chongqing Key Laboratory of Prevention and Treatment for Occupational Diseases and Poisoning, The First Affiliated Hospital of Chongqing Medical and Pharmaceutical College, Chongqing, China; ^5^ College of Public Health, Chongqing Medical University, Chongqing, China; ^6^ NHC Key Laboratory of Diagnosis and Treatment on Brain Functional Diseases, The First Affiliated Hospital of Chongqing Medical University, Chongqing, China

**Keywords:** hereditary spherocytosis, novel SPTB gene, mutation, hemolytic anemia, splenomegaly, jaundice

## Abstract

**Background:**

Hereditary spherocytosis (HS) is a group of genetically heterogeneous hereditary hemolytic disorders characterized by anemia, splenomegaly, jaundice, reticulocytosis, and spherical red blood cells on peripheral blood smears. Mutations in key genes, including *SPTB*, *ANK1*, *SLC4A1*, *SPTA1*, and *EPB42*, are commonly implicated in HS.

**Case Presentation:**

We report the case of a 22-year-old female presenting with anemia, jaundice, and a family history of splenectomy. Laboratory investigations revealed hemolytic anemia, elevated bilirubin levels, and peripheral blood smear findings consistent with HS. Genetic testing identified a novel *SPTB* gene splicing mutation (NM_001355436.2: c.1645-1G>A), inherited maternally, which is predicted to disrupt normal RNA splicing and protein synthesis.

**Discussion:**

The identified *SPTB* mutation expands the known mutation spectrum of the *SPTB* gene and highlights its role in the pathogenesis of HS. Clinical findings, combined with genetic analysis, confirmed the diagnosis of HS and underscored the importance of comprehensive molecular testing for accurate diagnosis, especially in patients with a strong family history.

**Conclusion:**

This case emphasizes the utility of genetic testing in diagnosing hereditary spherocytosis, particularly for novel gene mutations. Early and accurate molecular diagnosis facilitates better clinical management, family counseling, and treatment decisions for patients with HS.

## 1 Introduction

Hereditary spherocytosis (HS) is a genetically diverse group of hereditary hemolytic disorders characterized by various degrees of anemia, splenomegaly, jaundice, and the presence of spherocytes on peripheral blood smears (Nguyen et al., 2024). Patients with HS can experience a wide range of clinical symptoms, from mild anemia to severe hemolytic episodes requiring medical intervention (Qin et al., 2025). The underlying cause of HS is the structural weakness of the red blood cell membrane, which leads to its premature destruction in the spleen (Dogru et al., 2025). HS affects individuals across all age groups, with the disease manifesting at any point from early childhood to adulthood, depending on its severity (Bui et al., 2024). Although the disorder is well-documented in regions such as Scandinavia (*e.g.*, Norway, Sweden, and Denmark), where its prevalence is relatively high, it remains a rare condition in other parts of the world (*e.g.*, China), with an incidence of approximately 1.37 per 100,000 individuals in certain populations (Tian et al., 2023).

HS is genetically heterogeneous, arising from mutations in several different genes that encode proteins involved in the red blood cell membrane’s structural integrity (Da Costa et al., 2013; Russo et al., 2020). The majority of HS cases are inherited in an autosomal dominant manner, although autosomal recessive inheritance has also been documented. Mutations in genes such as *ANK1*, *SPTB*, *SLC4A1*, and *EPB42* are commonly associated with HS, and they affect the proteins ankyrin (Gariballa et al., 2024), β-spectrin (Boguslawska et al., 2021), band 310 (Liao et al., 2025), and protein 4.211, respectively (Muley et al., 2019). These mutations result in a weakened red blood cell membrane, which leads to the characteristic spherical shape and subsequent hemolysis (Delaunay, 2002). Among these, mutations in the *SPTB* gene, which encodes β-spectrin, have been identified as a significant contributor to HS (Jiang et al., 2023; Chi et al., 2024). Expanding the mutation spectrum of these genes is crucial for improving the diagnosis and management of the disease (Varadi et al., 2023; Qin et al., 2025).

In our clinical study, we found a case of HS caused by a novel mutation in the *SPTB* gene: the 22-year-old female patient exhibited clinical symptoms including jaundice and anemia, with a family history of splenectomy due to anemia. Laboratory findings confirmed hemolytic anemia with elevated levels of bilirubin. Genetic testing revealed a novel *SPTB* gene mutation, specifically NM_001355436.2:intron12.1645-1G>A, which was inherited from the patient’s mother. This novel mutation contributes to the growing body of evidence regarding the genetic heterogeneity of HS and highlights the importance of genetic testing in confirming the diagnosis, especially in regions where the condition is rare.

In addition, the identification of novel mutations such as the one presented in this case is important not only for improving our understanding of HS but also for expanding the known mutation spectrum of the *SPTB* gene (Mekonnen et al., 2024; Wang et al., 2024). This, in turn, provides valuable information for future genetic counseling, family planning, and personalized medical management of individuals affected by HS (Ge et al., 2023). Furthermore, the case underscores the role of genetic testing in diagnosing HS, particularly in atypical or mild presentations where the clinical symptoms may be less definitive. By documenting this case, we aim to contribute to the existing knowledge of HS and support clinicians in recognizing and diagnosing this rare genetic disorder.

## 2 Case history/examination

### 2.1 Case history

We conducted a retrospective analysis of a 22-year-old female patient who was admitted to our hospital on 21 October 2023. A comprehensive evaluation of her clinical history, physical examination findings, laboratory investigations, and genetic analyses was undertaken to determine the underlying causes of her elevated serum bilirubin levels and anemia.

### 2.2 Clinical assessment

The patient’s medical history revealed a cholecystectomy performed 6 years prior, necessitated by gallbladder stones. Particular attention was also given to her family medical history, which included her mother’s splenectomy for anemia. This thorough documentation provided crucial insights into potential hereditary factors contributing to the patient’s clinical presentation.

### 2.3 Physical examination

A comprehensive physical examination was performed upon admission, evaluating vital signs and signs of jaundice. The abdomen was assessed for tenderness, hepatomegaly, and splenomegaly, and peripheral edema was also noted.

## 3 Methods (differential diagnosis, investigations, and treatment)

### 3.1 Complete blood count

A complete blood count (CBC) was conducted using standard laboratory techniques to evaluate the following parameters: white blood cell (WBC) count, hemoglobin concentration, erythrocyte count, hematocrit, platelet count, and red blood cell distribution width (RDW). Blood samples were collected via venipuncture into sterile tubes containing EDTA as an anticoagulant. The samples were analyzed using an automated hematology analyzer (Abbott Cell-Dyn 3200 Automated Hematology Analyzer), which employed electrical impedance and light scattering methods for accurate cell counting and sizing. Quality control measures were implemented to ensure the reliability of results, and data were recorded for further interpretation of the patient’s hematological profile.

### 3.2 Liver function tests

Serum liver function tests were performed to assess the patient’s hepatic function and evaluate potential hemolytic processes by measuring total and unconjugated bilirubin levels. Blood samples were collected via venipuncture into sterile tubes, with serum separated by centrifugation at 3000 rpm for 10 min. The serum was then analyzed using automated biochemistry analyzers (Roche cobas^®^ 6000 analyzer) to determine bilirubin concentrations through colorimetric assays, following the manufacturer’s protocols. Quality control measures were employed to ensure accuracy and reliability of the results, which were subsequently interpreted in the context of the patient’s clinical presentation.

### 3.3 Anemia and hemolysis testing

A series of tests were conducted to evaluate the patient’s anemia and hemolysis, including measurements of vitamin B12, folic acid, and ferritin levels. Blood samples were collected via venipuncture, and serum was separated for analysis using automated immunoassays to quantify vitamin and ferritin levels. Peripheral blood smear analysis was performed by spreading a drop of blood on a microscope slide, staining with Wright–Giemsa stain, and examining the distribution of blood cell types under a light microscope. Direct and indirect antiglobulin tests were conducted to rule out autoimmune hemolytic anemia, following standard laboratory protocols. Additionally, an abdominal ultrasound was performed to assess splenic size and morphology, providing further insight into the underlying causes of anemia.

### 3.4 Bone marrow smear and bone marrow biopsy preparation

Bone marrow aspiration was performed using a sterile technique, inserting a 20-gauge needle into the posterior iliac crest under local anesthesia to collect approximately 1–2 mL of bone marrow aspirate into a sterile EDTA tube to prevent coagulation. A drop of the aspirated bone marrow was then placed on a clean glass microscope slide, and using another slide, a thin film was created by holding it at a 30°C–45°C angle and spreading the drop in a gentle, swift motion to form a uniform smear, which was allowed to air-dry completely at room temperature. The dried smear was fixed in 95% ethanol for 5 min, followed by staining using Wright–Giemsa stain, which involved applying Wright stain for 3–5 min, rinsing with buffered water for 1–2 min, and counterstaining with Giemsa stain for an additional 5–10 min. The slide was then rinsed with distilled water and air-dried before the prepared smear was examined under a light microscope at various magnifications (e.g., 100x and 400x), where hematopoietic cell types, morphology, and any abnormalities were assessed and documented.

A bone marrow biopsy was performed on the patient to obtain tissue samples for histopathological evaluation, following standard sterile techniques. Under local anesthesia, a trephine needle was inserted into the posterior iliac crest to extract a core of bone marrow. The biopsy specimens were then immediately placed in formalin for fixation. After 24 h of fixation, the samples were processed and embedded in paraffin wax, followed by sectioning at a thickness of 4–5 μm. The sections were mounted on glass slides and subjected to hematoxylin and eosin (HE) staining to assess cellular morphology and architectural features. The stained slides were examined under a light microscope at various magnifications to evaluate hematopoietic cell populations, assess any abnormalities, and comprehensively document the findings.

### 3.5 Gene sequencing

Genetic testing was performed to identify potential hemoglobinopathies and inherited mutations, including an analysis of the *SPTB* gene to detect any mutations, particularly the novel intron 12 c.1645-1G>A alteration. The detection method employed was next-generation sequencing (NGS) technology, performed on the Illumina sequencing platform. The detection scope included testing for over 2000 genes associated with hereditary blood, immune, and metabolic diseases; tumor susceptibility; and common single-gene hereditary disorders. Specifically, the analysis encompassed (1) point mutations (SNVs): single nucleotide variants, which are changes involving a single base pair in the DNA sequence; (2) small insertions and deletions (indels): small additions or losses of nucleotides within the DNA sequence; (3) specific intronic regions: non-coding regions adjacent to genes that may affect gene splicing or regulation; and (4) copy number variations (CNVs): large genomic alterations involving gains or losses of DNA segments. The analysis covered the whole exome and adjacent regions. The reference genome used was GRCh37/hg19, and the analysis was conducted using software tools such as fastp, BWA, SAMtools, GATK, VEP, CNVkit, and AnnotSV. Reference databases included ClinVar, HGMD, 1000 Genomes, DGV, gnomAD, and ExAC.

### 3.6 Pathogenicity assessment

The identified genetic variation was classified according to the American College of Medical Genetics and Genomics (ACMG) guidelines. Genetic testing was also conducted on the patient’s parents to establish inheritance patterns.

### 3.7 Treatment protocol

Based on the diagnostic findings, a treatment plan was formulated that included hepatoprotective agents (silymarin), medications to reduce jaundice (ursodeoxycholic acid), and medications to promote hematopoiesis (Shenghaibao Combination).

## 4 Results and conclusion

### 4.1 Clinical information

#### 4.1.1 Patient history

The patient, a 22-year-old female, presented with elevated serum bilirubin levels persisting for 3 days, accompanied by symptoms of jaundice and fatigue. Her medical history was notable for a cholecystectomy performed 6 years earlier due to gallbladder stones, with no history of recurrent gallbladder or biliary issues post-surgery. She denied any prior blood transfusions, chronic illnesses, or significant medication use.

Family history revealed that the patient’s mother had undergone a splenectomy for anemia, suggesting a possible hereditary basis for her condition. There was no reported family history of gallstones, jaundice, or other hematologic or metabolic disorders affecting other relatives. Additionally, the patient’s psychosocial history indicated no significant stressors, environmental exposures, or occupational risks. She had no history of smoking, alcohol consumption, or drug use (Table 1).

**TABLE 1:** Timeline of key clinical events and interventions.

Date	Event	Details
6 years ago	Cholecystectomy	Patient underwent gallbladder removal due to gallstones
Oct-23	Presentation of symptoms	Patient presented with anemia and elevated serum bilirubin levels
21-Oct-23	Admission to hospital	Patient admitted for further evaluation of anemia and jaundice
22-Oct-23	Laboratory investigations	Confirmed anemia, hyperbilirubinemia, and other abnormalities
23-Oct-23	Genetic testing conducted	Identified novel c.1645-1G>A mutation in the *SPTB* gene
24-Oct-23	Diagnosis of HS	Based on genetic testing and clinical features
25-Oct-23	Start of treatment	Initiated oral hemopexin, silymarin, and ursodeoxycholic acid
30-Oct-23	Discharge and follow-up plan	Patient discharged with outpatient follow-up recommendations

#### 4.1.2 Physical examination

On physical examination, the patient’s vital signs were within normal limits (blood pressure: 110/70 mmHg, heart rate: 76 bpm, respiratory rate: 18 breaths/min, and temperature: 36.8°C). She appeared alert and oriented. The most notable clinical finding was a mild icteric tinge observed on the face, accompanied by generalized jaundice affecting the skin, mucosa, and sclera. There were no signs of cyanosis, clubbing, or petechiae. Peripheral lymph nodes were non-palpable, and no lymphadenopathy was detected.

Abdominal examination revealed a flat, non-distended abdomen with no tenderness, rebound pain, or muscle guarding. Hepatomegaly was not appreciable upon palpation, but the spleen was palpable approximately 2 cm below the left costal margin, firm but non-tender. There was no evidence of ascites or peripheral edema. Cardiovascular, respiratory, and neurological examinations were unremarkable. Objective measures from clinical observations included a total bilirubin level of 6.8 mg/dL, with direct bilirubin at 4.2 mg/dL. Hemoglobin levels were low at 8.6 g/dL, consistent with anemia, while other vitals and laboratory parameters were within normal ranges, except for mild reticulocytosis.

### 4.2 Laboratory investigations

#### 4.2.1 Complete blood count

A CBC revealed the following results: (1) WBC count: 10.57 × 10^9^/L (↑; reference range: 4.0–10.0 × 10^9^/L); (2) neutrophil count: 6.96 × 10^9^/L (within normal limits; reference range: 1.5–7.0 × 10^9^/L); (3) erythrocyte count: 2.97 × 10^12^/L (↓; reference range: 3.8–5.1 × 10^12^/L); (4) Hgb concentration: 96.00 g/L (↓; reference range: 120–160 g/L); (5) Hct: 29.9% (↓; reference range: 36%–48%); (6) red blood cell distribution width (RDW-CV): 22% (↑; reference range: 11.5%–14.5%); (7) RDW standard deviation: 71.2 fL (↑; reference range: 39–46 fL); and (8) platelet count: 230.00 × 10^9^/L (within normal limits; reference range: 150–400 × 10^9^/L).

The elevated WBC count (10.57 × 10^9^/L) suggests a possible underlying inflammatory or immune response, which may be related to the hemolytic process observed in this patient. However, given that the neutrophil count was within the normal range (6.96 × 10^9^/L), the elevated WBC count is unlikely to indicate a significant bacterial infection but may be associated with the systemic inflammatory response secondary to hemolysis or splenomegaly.

#### 4.2.2 Liver function tests

Liver function tests revealed elevated total bilirubin at 128.55 μmol/L (↑; reference range: 5–21 μmol/L) and unconjugated bilirubin at 112.80 μmol/L (↑; reference range: 1.7–17.0 μmol/L), suggesting ongoing hemolysis and impaired bilirubin processing.

#### 4.2.3 Hematological and anemia-related tests

Key markers for anemia revealed (1) vitamin B12: 273 pg/mL (within normal range; reference range: 200–900 pg/mL); (2) folic acid: 7.03 ng/mL (within normal range; reference range: 3.0–17.0 ng/mL); and (3) ferritin: 560 ng/mL (↑; reference range: 20–200 ng/mL), suggesting adequate iron stores.

Peripheral blood smear analysis demonstrated (1) neutrophilic segmented granulocytes: 70% (within normal range; reference range: 40%–70%); (2) mature lymphocytes: 17% (within normal range; reference range: 20%–40%); and (3) Mature monocytes: 8% (within normal range; reference range: 2%–8%).

#### 4.2.4 Hemolysis-related investigations

Direct and indirect antiglobulin tests (Coombs tests): both negative, ruling out autoimmune hemolytic anemia.

Abdominal ultrasound revealed splenomegaly, with the spleen measuring 141 mm × 59 mm, consistent with the hemolytic process and indicative of moderate enlargement.

#### 4.2.5 Bone marrow morphology

**FIGURE 1:**
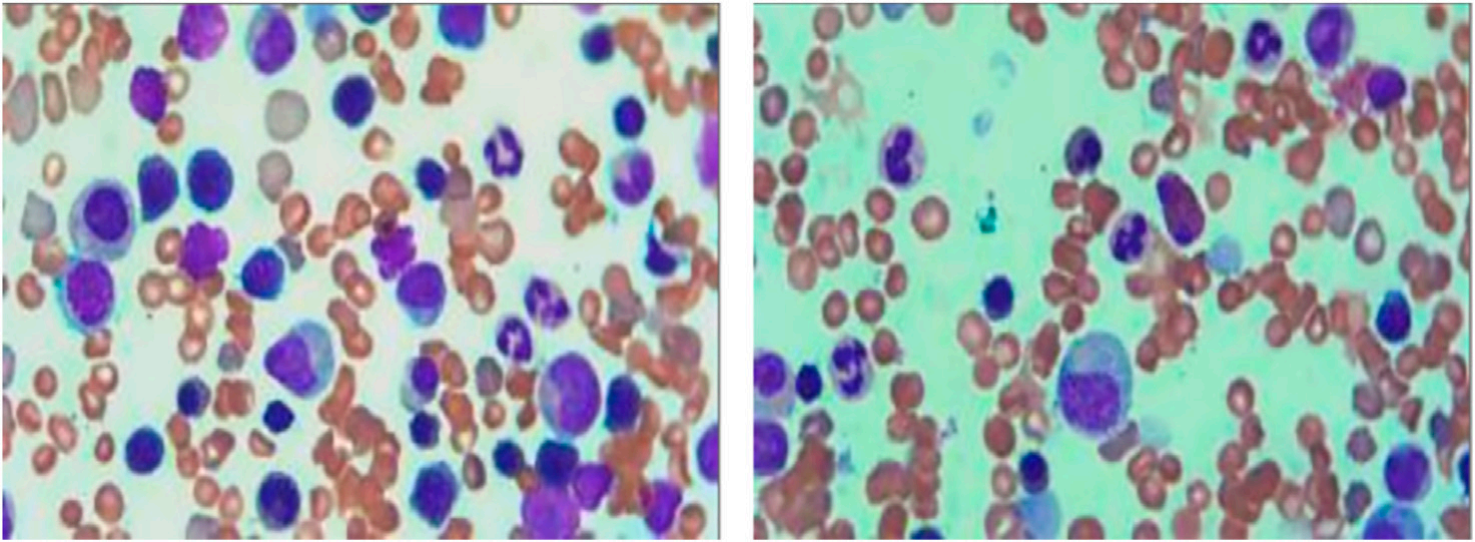
Bone marrow smear of the patient. The bone marrow smear shows a predominance of erythroid precursors with a reversal of the granulocyte-to-erythrocyte (G/E) ratio, indicative of erythroid hyperplasia. The presence of dysmorphic erythroblasts and immature forms suggests an underlying hemolytic process.

Bone marrow aspirate (from the posterior iliac crest) indicated proliferative anemia morphology with an inversion of the granulocyte-to-erythrocyte (G/E) ratio, further supporting the diagnosis of HS (Figure 1).

#### 4.2.6 Bone marrow biopsy findings

**FIGURE 2:**
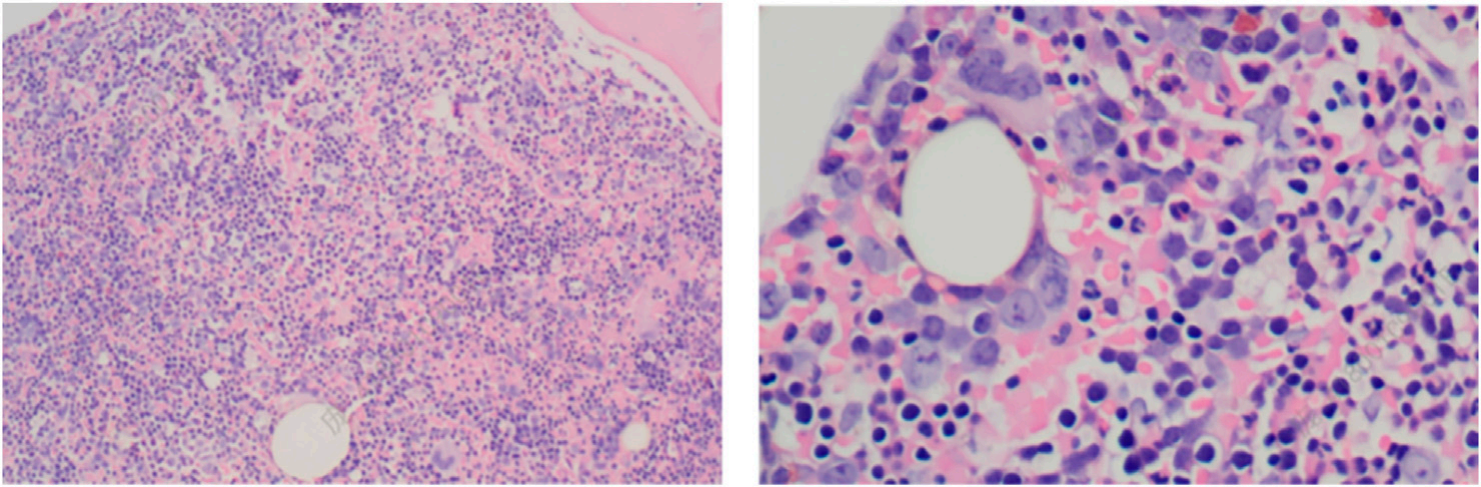
Bone marrow biopsy of the patient (HE staining). The bone marrow biopsy reveals hypercellularity with erythroid predominance, further supporting the diagnosis of hemolytic anemia. The erythroid hyperplasia and reduction in granulocytic elements demonstrate a disrupted hematopoietic balance, characteristic of conditions with increased erythropoietic demand.

Pathological analysis of the bone marrow biopsy revealed the following:

1. Hematopoietic tissue: extremely active hematopoietic tissue hyperplasia, constituting over 90% of marrow volume, with a marked decrease in adipose tissue.
2. Granulocytic series: proliferative, with immature precursor cells scattered throughout. Cells in the middle and late promyelocyte stage and below predominated, with no significant morphological abnormalities.
3. Erythroid series: hyperplastic, with an increased proportion of erythroblasts. Cells in the middle and late erythroblast stages were most prevalent, without obvious morphological abnormalities.
4. Megakaryocytes: present at a density of 0–5 per high-power field (HPF), with no abnormalities in size or morphology.
5. Lymphocytes and plasma cells: scattered throughout the sample.
6. Fibrous tissue: minimal areas of scattered fibrotic hyperplasia (Figure 2).

#### 4.2.7 Genetic testing for hemoglobinopathies

Thalassemia genetic analysis: no detectable abnormalities were found in the α-thalassemia gene (deletion and point mutation types) or β-thalassemia genotype analysis.

#### 4.2.8 Genetic analysis

Genetic testing identified a novel variant in the *SPTB* gene (NM_001355436.2), specifically an intron 12 c.1645-1G>A alteration. This mutation occurs at the splice acceptor site, involving a single-nucleotide substitution from guanine (G) to adenine (A) at the position one base pair upstream of nucleotide 1645 in the cDNA sequence of the *SPTB* gene. Such a mutation is predicted to disrupt normal RNA splicing, potentially resulting in abnormal protein synthesis. This molecular abnormality aligns with the patient’s clinical features of HS, such as anemia, reticulocytosis, hyperbilirubinemia, and the presence of spherocytes on the peripheral blood smear (Qin et al., 2025).

The *SPTB* gene was specifically targeted for testing based on the patient’s clinical presentation and its well-established association with HS (Tang et al., 2024). In addition to *SPTB*, other known HS-related genes—*SLC4A1*, *ANK1*, *SPTA1*, and *EPB42*—were also included in the genetic analysis through targeted sequencing (Da Costa et al., 2013). However, no pathogenic or likely pathogenic variants were identified in these genes, further highlighting the causative role of the *SPTB* c.1645-1G>A variant in this case.

Interestingly, sequencing also revealed a variant in the *KRIT1* gene, a gene associated with cerebral cavernous malformations (CCM, OMIM: 116860) and, in rare cases, hyperkeratotic capillary-venous malformations (Nguyen et al., 2024). Despite its detection, the *KRIT1* variant has no known association with HS and is considered an incidental finding. It is unrelated to the patient’s current clinical presentation and holds no clinical relevance to the diagnosis of HS in this case (Tables 2, 3).

**TABLE 2:** Information on patient’s genetic variants.

Serial number	Gene	Mutation information	Mutation-site region reference sequence
1	*KRIT1*	c.1355_1358dup:p.Q455Lfs*26	TTT​CTT​G[AGAG]AGA​CGC​ATT​C
2	*SPTB*	c.1645-1G>A	AGA​GGT​GAG​C[C]TGG​CAA​AGA​G

**TABLE 3:** Genetic test results of the patient.

Gene	Chromosome location^(1)^	Mutation information	Genotype^(2)^	PopFreqMax^(3)^	REVEL ^(4)^	Related Diseases (OMIM; inheritance)^(5)^	ACMG categorization ^(6)^
*SPTB*	chr14:65261336C>T	NM_001355436.2:intron12:c.1645-1G>A	Hybridization	-	-	Elliptocytosis type 3 (617948); spherocytosis type 2 (616649; AD)	LP

Note: 1. The chromosomal position is referenced to the GRCh37/hg19 genome. 2. The genotype ‘Hom’ indicates a homozygous variant at this locus, ‘Het’ indicates a heterozygous variant, ‘Hemi’ signifies a hemizygous variant, ‘WT’ (wild-type) denotes no variation at this locus, and ‘NA’ indicates unknown status. 3. PopFreqMax represents the maximum allele frequency of this variant in normal population databases, primarily in gnomAD_genome_ALL and gnomAD_genome_EAS. 4. REVEL is a protein function prediction tool, with values closer to ‘1’ indicating a potentially greater impact of the variant on protein function. 5. Relevant diseases are referenced from the OMIM database; ‘AD’ indicates autosomal dominant inheritance, ‘AR’ indicates autosomal recessive inheritance, ‘XLD’ denotes X-linked dominant inheritance, ‘XLR’ indicates X-linked recessive inheritance, ‘YL’ refers to Y-linked inheritance, and ‘NA’ signifies unknown inheritance mode. 6. The ACMG classification is based on the guidelines established by the American College of Medical Genetics and Genomics (ACMG) and the American Society for Clinical Pathology (AMP) in 2015 for the interpretation of genetic variants, the general guidelines from the ClinGen Sequence Variant Interpretation Working Group (ClinGen SVI), and recommendations from the ClinGen Variant Curation Expert Panel (ClinGen VCEP). The classification is divided into five categories, namely, pathogenic (P), likely pathogenic (LP), uncertain significance (VUS), likely benign (LB), and benign (B).

#### 4.2.9 Pathogenicity assessment

**FIGURE 3:**
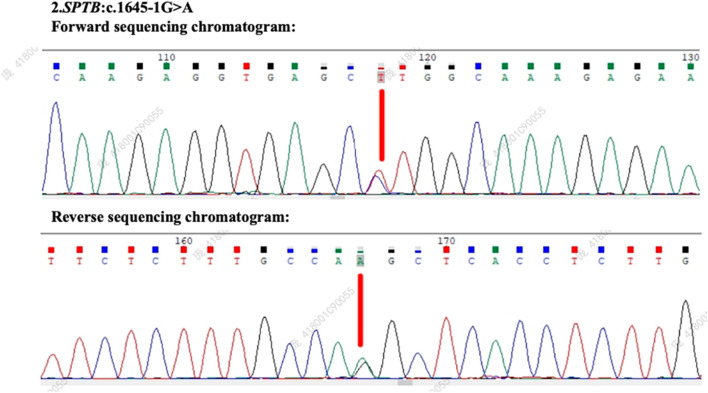
Genetic testing results and sequencing chromatogram of the patient’s mother. The forward and reverse sequencing chromatograms demonstrate the presence of the *SPTB* c.1645-1G>A mutation in the patient’s mother (indicated by the red arrow). This variant affects the splice acceptor site of intron 12, supporting the maternal inheritance of the mutation.

**FIGURE 4:**
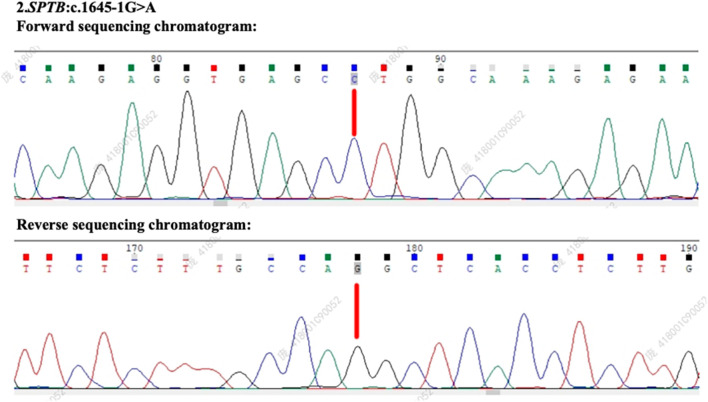
Genetic testing results and sequencing chromatogram of the patient’s father. The forward and reverse sequencing chromatograms reveal the absence of the *SPTB* c.1645-1G>A mutation in the patient’s father. This confirms that the variant identified in the patient was maternally inherited.

Based on the ACMG guidelines, the identified *SPTB* gene variation (NM_001355436.2: intron 12: c.1645-1G>A) has been preliminarily classified as likely pathogenic, supported by criteria PVS1 (null variant in a gene where loss of function is a known mechanism of disease) and PM2 (absence in population databases). Following patient consent, genetic testing was performed on both parents. The *SPTB* c.1645-1G>A variant was found to be positive in the patient’s mother (Figure 3), whereas the patient’s father tested negative for this mutation (Figure 4), suggesting maternal inheritance of the variant.

### 4.3 Diagnostic assessment

The patient’s diagnostic journey was complex and required careful consideration of various factors. Her family history was significant as her mother had undergone a splenectomy due to chronic anemia, raising suspicion of a hereditary hematologic condition. On admission, physical examination revealed splenomegaly, jaundice, and signs consistent with hemolytic anemia. Laboratory tests confirmed anemia with a hemoglobin level of 8.6 g/dL, elevated total bilirubin (6.8 mg/dL), and an unconjugated bilirubin predominance. Both direct and indirect antiglobulin (Coombs) tests were negative, ruling out autoimmune hemolytic anemia.

Genetic testing was conducted due to the hereditary implications suggested by the family history and clinical presentation. Testing identified a novel mutation in the *SPTB* gene, consistent with HS. This diagnosis was favored over other potential conditions, such as thalassemia, based on the absence of microcytosis, normal hemoglobin electrophoresis, and the presence of spherocytes on the peripheral blood smear.

Challenges in the diagnostic process included differentiating HS from other hereditary anemias and addressing the patient’s concurrent liver dysfunction. Elevated liver enzymes upon admission added complexity, raising concerns about possible hemolysis-induced hepatic stress or an underlying liver condition. Despite these challenges, a multidisciplinary approach integrating clinical findings, laboratory tests, and genetic analysis led to a definitive diagnosis of HS. This highlights the importance of a comprehensive diagnostic process in such cases.

Prognostically, HS is associated with chronic hemolysis and complications such as gallstone formation and splenomegaly, both of which were relevant in this case. Early diagnosis and management are crucial in improving the patient’s quality of life and preventing long-term complications.

### 4.4 Therapeutic intervention

The therapeutic approach was tailored to address the patient’s symptoms and underlying condition. Upon admission, the patient exhibited significant anemia and jaundice, warranting immediate and targeted intervention. Initial treatment included 1. silymarin: administered to support liver function and protect hepatocytes from damage caused by hemolysis-related oxidative stress; 2. ursodeoxycholic acid: used to alleviate jaundice and prevent bile acid toxicity by promoting bile flow and reducing bilirubin levels; and 3. Shenghaibao Combination: a traditional Chinese medicine preparation prescribed to promote hematopoiesis and enhance red blood cell production.

The rationale for these interventions was grounded in the dual goals of addressing the patient’s acute symptoms and preventing further complications. Regular monitoring of the patient’s hemoglobin levels, bilirubin levels, and liver function was undertaken to assess therapeutic efficacy and guide adjustments to the treatment plan.

As the patient’s condition stabilized, additional interventions were discussed, including the potential long-term benefits of splenectomy for managing hemolysis and reducing splenomegaly. However, this was deferred due to the patient’s current stability and the preference for conservative management at this stage.

### 4.5 Follow-up and outcomes

The patient was followed closely during and after hospitalization to monitor her response to treatment. Over the course of 2 months, her bilirubin levels and hemoglobin levels gradually improved, with total bilirubin decreasing to 2.4 mg/dL and hemoglobin stabilizing at 11.2 g/dL. Liver enzyme levels normalized with silymarin therapy. The patient demonstrated good adherence to the prescribed interventions, attending all follow-up appointments and adhering to dietary and lifestyle recommendations to minimize hemolytic stress.

No significant adverse events were reported during follow-up, although the patient occasionally experienced mild fatigue, which resolved with rest. Ultrasound imaging at follow-up confirmed that splenomegaly persisted but remained stable without signs of hypersplenism.

Future plans include continued conservative management with periodic monitoring of hematologic parameters, and the option of splenectomy will be revisited if symptoms worsen or complications arise.

### 4.6 Patient perspective

The patient shared her perspective on her condition and treatment, describing initial anxiety and fear upon learning of her diagnosis. She expressed concern about the hereditary nature of her condition and its potential impact on her future health. However, she felt reassured by the comprehensive diagnostic process and the support provided by her medical team.

The patient reported significant improvement in her symptoms following treatment, with a marked reduction in jaundice and an improvement in her overall energy levels. She expressed appreciation for the personalized approach to her care, which combined both conventional and traditional therapies.

Looking ahead, the patient is optimistic about managing her condition and has expressed gratitude for the guidance and education provided during follow-up visits. She feels empowered to maintain her health and adhere to the recommended treatment plan, emphasizing the importance of continued collaboration with her medical team.

## 5 Discussion

HS is primarily attributed to molecular defects in the erythrocyte membrane skeleton, resulting from mutations in genes encoding critical membrane proteins (Russo et al., 2020). These mutations disrupt the structural stability of the erythrocyte membrane-skeletal network, reducing deformability (Polizzi et al., 2025). Consequently, erythrocytes lose mechanical stability, undergo splenic sequestration, and are prematurely destroyed (Qin et al., 2025). Extensive research has identified key mutations in genes such as SLC4A1, EPB42, SPTA1, SPTB, and ANK1 (Da Costa et al., 2013; Russo et al., 2020). These mutations lead to defects in associated membrane proteins—such as band 3 (Tian et al., 2023), protein 4.2 (Cabiati et al., 2024), α-spectrin (Vercellati et al., 2022), β-spectrin Panizo Morgado et al., 2020), and ankyrin (Lazzareschi et al., 2019)—altering the morphology of erythrocytes from a biconcave disc to a spherical shape, a hallmark of HS (More et al., 2023).

The genetic landscape of HS varies across populations. Studies indicate that SPTA1, ANK1, and SPTB are the most commonly mutated genes in global HS cohorts (Shi et al., 2023; Tian et al., 2023; Allard et al., 2024; Gariballa et al., 2024). In contrast, domestic investigations in China reveal ANK1 and SPTB as the predominant mutations among Chinese HS patients (Li et al., 2022; Vercellati et al., 2022), underscoring racial heterogeneity and variability in genetic predispositions (Yamamoto et al., 2022; Donaty et al., 2024). Nonsense and frameshift mutations are the most frequently reported pathogenic variants (Gou et al., 2024; He et al., 2024; Panarach et al., 2024). In this case, a novel heterozygous mutation (c.1645-1G>A) in the SPTB gene was identified. This mutation, located at the splice acceptor site in intron 12, results in a guanine-to-adenine substitution and is deemed causative based on its co-segregation with the disease in the patient’s mother, who harbors the same variant (Jiang et al., 2023; Kang et al., 2023; Zhou et al., 2023).

The SPTB gene, located on chromosome 14q23–q24.2, encodes β-spectrin, a critical cytoskeletal protein involved in maintaining erythrocyte membrane integrity through interactions with ankyrin and other cytoskeletal components (Liu et al., 2020; Xi et al., 2023; Tang et al., 2024; Dogru et al., 2025; Qin et al., 2025). Mutations in SPTB have been implicated in a spectrum of hereditary red blood cell disorders, including HS type 2, hereditary elliptocytosis, and neonatal hemolytic anemia (Llaudet-Planas et al., 2018; Andolfo et al., 2021; Songdej et al., 2024). The identification of a novel pathogenic variant in SPTB enriches the genetic database for HS and highlights the role of genetic analysis in expanding our understanding of this disease.

Diagnosing HS remains a challenge due to its heterogeneous clinical presentation and the lack of universally accepted diagnostic markers (Chi et al., 2024; Habibzadeh et al., 2024; Melo et al., 2024). Although the osmotic fragility test has historically been the gold standard for HS diagnosis (Pereira Garcia et al., 2024), it is limited by suboptimal sensitivity and specificity (Rey-Barroso et al., 2023). Advances in diagnostic techniques, such as the eosin-5′-maleimide (EMA) binding test (Uman et al., 2022), flow cytometric osmotic fragility testing (Azoulay et al., 2022), and laser diffraction analysis (Pal et al., 2024), have significantly improved diagnostic accuracy. Moreover, NGS has revolutionized the field by enabling precise identification of pathogenic variants in HS-associated genes (Peng et al., 2024; Sotiriou et al., 2024). In this case, NGS identified a novel SPTB mutation, confirming the diagnosis of HS and exemplifying its utility in cases with atypical presentations or inconclusive traditional test results.

A thorough investigation of family history is critical in diagnosing HS, as genetic inheritance plays a pivotal role (Casabianca et al., 2024; Liang et al., 2024; Xiong et al., 2024). The identification of the same SPTB mutation in the patient’s mother supports the pathogenicity of the variant and underscores the importance of genetic counseling (Tang et al., 2024; Tognetti et al., 2024; Qin et al., 2025). However, in patients without a family history, differential diagnosis should exclude other causes of hemolytic anemia through osmotic fragility testing and genetic analysis to ensure a definitive diagnosis (Orzan et al., 2023; Shi et al., 2023; Tian et al., 2023; Allard et al., 2024).

Treatment of HS depends on disease severity. Although red blood cell transfusions and splenectomy remain standard interventions for severe cases (Soldin et al., 2024; Yabe et al., 2024), mild cases, such as that of our patient, are typically managed conservatively. This involves routine monitoring of hemoglobin and bilirubin levels, liver function, and regular ultrasound assessments of the liver and spleen (Tognetti et al., 2024; Qin et al., 2025). For mild to moderate anemia, transfusions may be necessary, especially in the presence of complications such as infections (Fermo et al., 2021; Li et al., 2022). In this case, the patient received supportive care with oral hemopexin, silymarin, and ursodeoxycholic acid to optimize erythrocyte turnover and liver function. The patient was discharged with a plan for regular outpatient follow-up to ensure ongoing monitoring.

This case highlights several important considerations for clinical practice and research. First, the identification of a novel SPTB variant expands the known genetic landscape of HS and underscores the value of genetic testing in diagnosing rare variants. Second, the findings emphasize the need for clinicians to remain vigilant for atypical presentations of HS and use a multidisciplinary approach, including advanced diagnostic techniques and genetic counseling, to achieve accurate diagnosis and management. Third, the case demonstrates the importance of considering racial and genetic heterogeneity when developing diagnostic and therapeutic strategies.

From a broader perspective, this case adds to the growing body of evidence supporting the integration of genetic testing into routine clinical practice for hereditary disorders (Li and Mao, 2024). Further research is needed to elucidate the functional impact of novel SPTB variants and explore targeted therapeutic strategies (Meglic et al., 2020; Nieminen et al., 2021). Additionally, as advances in precision medicine continue, there is potential to develop individualized management plans for HS patients based on their specific genetic profiles (Rey-Barroso et al., 2023; Rayamajhi et al., 2024).

In summary, this case demonstrates the clinical and diagnostic challenges associated with HS and highlights the role of genetic analysis in overcoming these hurdles. By integrating advanced diagnostic tools and a patient-centered approach, clinicians can improve diagnostic accuracy, optimize treatment outcomes, and contribute to the growing understanding of HS (Zhang et al., 2024).

## 6 Conclusion

This case report highlights the identification of a novel mutation in the *SPTB* gene (NM_001355436.2: intron 12 c.1645-1G>A) as the underlying cause of HS in a young female patient. This discovery adds to the growing mutation spectrum of the *SPTB* gene, enhancing our understanding of its role in the pathogenesis of HS and reinforcing the genetic heterogeneity of the disease.

Our findings emphasize the critical role of genetic testing in diagnosing HS, especially in regions where the condition is uncommon or presents atypically. Early and accurate identification of genetic mutations not only aids in confirming the diagnosis but also provides essential information for genetic counseling and tailored management strategies. By documenting this novel mutation, we contribute valuable knowledge to the field, which may improve diagnostic accuracy and personalized care for individuals with HS in the future.

Key clinical message: a novel mutation in the *SPTB* gene (NM_001355436.2:intron12.1645-1G>A) was identified in a 22-year-old female with HS, highlighting the importance of genetic testing in confirming diagnoses of rare genetic disorders, expanding the mutation spectrum, and guiding personalized treatment in typical cases.

## Data Availability

The original contributions presented in the study are included in the article, further inquiries can be directed to the corresponding author.
